# Prehabilitation in Colorectal Cancer Surgery: A Narrative Review of Current Evidence and Clinical Perspectives

**DOI:** 10.3390/nu18152457

**Published:** 2026-07-27

**Authors:** Dorota Zierkiewicz, Stanisław Manulik, Anna Chudiak, Dorota Krówczyńska, Wojciech Homola, Piotr Pobrotyn, Janecka Wioletta, Mariola Głowacka, Małgorzata Paprocka-Borowicz, Mariusz Chabowski

**Affiliations:** 1 Division of Anesthetic and Surgical Nursing, Faculty of Nursing and Midwifery, Wroclaw Medical University, 51-618 Wroclaw, Poland; dorota.zierkiewicz@umw.edu.pl (D.Z.); anna.chudiak@umw.edu.pl (A.C.); 2 Department of Gastrointestinal Cancer, Lower Silesian Oncology, Hematology and Pulmonology Center, 53-413 Wroclaw, Poland; 3 Division of Healthcare Organization, Department of Nursing, Faculty of Nursing and Midwifery, Wroclaw Medical University, 51-618 Wroclaw, Poland; stanislaw.manulik@umw.edu.pl; 4 Intensive Cardiac Care Unit, Department of Heart Failure and Transplantation, Cardinal Stefan Wyszynski Institute of Cardiology in Warsaw, 04-628 Warsaw, Poland; dkrowczynska@o2.pl; 5 Department of Nursing and Obstetrics, Collegium Mazovia, 08-110 Siedlce, Poland; 6 Department of Nursing, Faculty of Rehabilitation, Józef Piłsudski University of Physical Education in Warsaw, 00-968 Warsaw, Poland; 7 Gynaecology and Obstetrics Centre FemiMea, 55-040 Bielany Wroclawskie, Poland; wojtek.homola@gmail.com; 8 Department of Clinical Neurosciences, Faculty of Medicine, Wrocław University of Science and Technology, 51-377 Wroclaw, Poland; 9 Department of Nursing, Faculty of Health Sciences, The Mazovian University in Płock, 09-402 Płock, Poland; w.janecka@mazowiecka.edu.pl (J.W.); m.glowacka@mazowiecka.edu.pl (M.G.); 10 Department of Neurological Rehabilitation, Regional Specialist Hospital in Wroclaw, 51-128 Wroclaw, Poland; malgorzata.paprocka-borowicz@wssk.wroc.pl; 11 Department of Surgery, 4th Military Teaching Hospital in Wroclaw, 50-981 Wrocław, Poland; mariusz.chabowski@pwr.edu.pl; 12 Department of Clinical Surgical Sciences, Faculty of Medicine, Wroclaw University of Science and Technology, 51-377 Wroclaw, Poland

**Keywords:** colorectal cancer, surgical treatment, prehabilitation, perioperative care, enhanced recovery after surgery, state-of-the-art review

## Abstract

**Background/Objectives**: Surgical treatment of colorectal cancer (CRC) is associated with a substantial risk of postoperative complications and delayed recovery of functional capacity, particularly in older patients and those with multiple comorbidities. Preoperative prehabilitation has emerged as a strategy aimed at optimizing patients’ physical, nutritional, and psychological status prior to surgery; however, its clinical effectiveness and feasibility of implementation remain subjects of ongoing debate. The aim of this narrative review was to provide a clinically oriented synthesis of current evidence regarding multimodal prehabilitation in patients undergoing CRC surgery, with particular emphasis on perioperative outcomes, functional recovery, nutritional optimization, and implementation-related considerations. **Methods**: A targeted narrative review of the peer-reviewed literature indexed in PubMed (2015–2025) was conducted to identify contemporary review-type publications, including narrative reviews, systematic reviews, scoping reviews, and meta-analyses, addressing prehabilitation in CRC surgery. The findings were synthesized narratively and interpreted with emphasis on clinical applicability and translational relevance. **Results**: Twenty contemporary review publications were included in the analysis. Across the included publications, the most frequently reported favorable effects were observed for multimodal prehabilitation, incorporating physical exercise, nutritional support, and a psychological component. These interventions were associated with improvements in functional capacity and, in some meta-analyses, with a reduction in postoperative complication rates and shorter length of hospital stay, particularly in high-risk patients. Prehabilitation was assessed as safe and well tolerated. **Conclusions**: The current body of evidence indicates that multimodal prehabilitation may constitute a beneficial adjunct, particularly in improving functional capacity, while its impact on hard postoperative outcomes remains closely dependent on intervention quality, appropriate patient selection, and seamless integration with established perioperative care pathways. Future meta-analyses should address underexplored components of prehabilitation, including preparation for stoma formation and targeted educational and psychological support related to sexual health.

## 1. Introduction

Colorectal cancer (CRC) remains one of the most frequently diagnosed malignant neoplasms worldwide and constitutes a major cause of morbidity and high mortality in the general population. Global epidemiological data indicate that CRC is the second to third leading cause of cancer-related death and one of the most commonly diagnosed malignancies, with its incidence and clinical burden continuing to increase in parallel with population ageing and lifestyle changes [1].

CRC exhibits substantial clinical heterogeneity, and its radical treatment using oncologic surgery, despite advances in surgical techniques, remains associated with a high risk of perioperative and postoperative complications. These include surgical site infections, cardiopulmonary events, anastomotic healing failure, and the need for rehospitalization, which translate into prolonged hospital stays, deterioration in quality of life, and poorer long-term treatment outcomes [2]. A high incidence of postoperative complications following CRC surgery has been demonstrated in numerous cohort analyses and literature reviews, with frailty and comorbidities identified as significant predictors of adverse clinical outcomes in patients undergoing CRC resection [3].

Multimodal prehabilitation typically includes components of physical exercise and nutritional intervention and may be further expanded to incorporate psychological support, targeted functional interventions (e.g., respiratory training), and medical optimization (e.g., iron supplementation in the presence of anemia and smoking cessation) [4]. Compared with standard care and postoperative rehabilitation, evidence of moderate quality suggests that multimodal prehabilitation leads to improved postoperative functional recovery in many oncological patient populations [5].

In recent years, the concept of prehabilitation has been formally incorporated into modern perioperative care models as a complementary element of ERAS (Enhanced Recovery After Surgery) protocols, focusing on optimization of the patient’s condition prior to the initiation of surgical treatment [6,7]. According to current recommendations and expert guidelines, prehabilitation constitutes a comprehensive, interdisciplinary process encompassing improvement of nutritional status, enhancement of physical capacity, psychological support, elimination of harmful habits, and optimization of comorbid conditions, initiated from the time of qualification for treatment [8,9,10].

In a systematic review addressing elective surgical procedures, a positive effect of prehabilitation on patients’ physical fitness was confirmed; however, limited evidence was identified regarding its impact on postoperative clinical outcomes in candidates for elective cardiac surgery, abdominal surgery, and thoracic surgery [11]. Moreover, current studies describe beneficial effects of prehabilitation on treatment outcomes and reductions in healthcare costs among patients with CRC who underwent prehabilitation prior to planned colorectal surgery. Nevertheless, debate persists as to whether prehabilitation should be incorporated into standard care in colorectal surgery [12].

Current research is focused on adapting prehabilitation programs to the needs of specific patient populations undergoing surgical treatment, and expanding their geographical scope would enrich research efforts by providing valuable information for healthcare professionals shaping therapeutic strategies. The most recent literature analyses demonstrate an upward trend in the number of publications on prehabilitation (North America and Western Europe), particularly in fields such as surgery, rehabilitation, oncology, orthopedics, gastroenterology, and hepatology. Preoperative exercise remains the primary area of interest, while growing attention is being directed toward multimodal prehabilitation and its effectiveness based on the characteristics of specific patient groups [13].

In recent years, there has been increasing interest among researchers in publishing systematic reviews and meta-analyses synthesizing existing knowledge on the clinical application of prehabilitation in patients undergoing surgical treatment for CRC, as reflected by the growing body of review-level evidence included in this analysis. In the context of a growing patient population and increasing pressure on the economic efficiency of healthcare systems, multimodal prehabilitation represents an intervention with high implementation potential, particularly within systems based on comprehensive oncological care pathways.

In Central and Eastern European countries, the concept of multimodal prehabilitation remains at an early stage of implementation, which translates into limited access for oncological patients. Therefore, it is important to draw upon good practices and experiences from countries and healthcare systems that have successfully implemented prehabilitation solutions on a systemic level for years and have reported these efforts in numerous review publications, including both systematic reviews and meta-analyses.

The aim of this narrative review was to provide a comprehensive synthesis of current evidence regarding the effectiveness of prehabilitation (particularly multimodal interventions) in patients undergoing elective surgical treatment for CRC, with particular emphasis on its impact on the incidence of postoperative complications, functional capacity, length of hospital stay, and other clinically relevant treatment outcomes, as well as to identify key areas of uncertainty, methodological limitations, and directions for future research. In addition to physical exercise, nutritional optimization, and psychological support, contemporary multimodal prehabilitation increasingly encompasses patient-centred interventions such as stoma education, sexual health counselling, and shared decision-making, reflecting the broader concept of perioperative preparation.

## 2. Materials and Methods

### 2.1. Conceptual Framework

This article was designed as a narrative review aimed at providing a clinically oriented synthesis of current knowledge regarding prehabilitation in patients undergoing CRC surgery. The primary objective was not to perform a formal umbrella review or quantitative overview of reviews, but rather to integrate and critically interpret contemporary evidence in order to identify recurring themes, clinically relevant findings, implementation challenges, and future directions for research and practice.

Particular emphasis was placed on multimodal prehabilitation strategies incorporating exercise, nutritional optimization, and psychological support, as well as on their potential role within contemporary perioperative care pathways, including ERAS programmes.

### 2.2. Source of Evidence

The narrative synthesis was informed by contemporary peer-reviewed literature published between 2015 and 2025. The evidence base was derived primarily from review-level publications, including systematic reviews, meta-analyses, scoping reviews, and selected narrative reviews addressing prehabilitation in CRC surgery or closely related oncological surgical populations.

PubMed served as the principal source of literature retrieval because of its broad coverage of biomedical and clinical research. Additional publications identified through reference tracking of key articles were considered when relevant to the scope of the review. 

Priority was given to contemporary peer-reviewed publications providing comprehensive evidence on clinically relevant aspects of prehabilitation in CRC surgery, including functional outcomes, perioperative recovery, implementation considerations, and patient-centered care.

### 2.3. Narrative Review

Given the heterogeneity of prehabilitation interventions, patient populations, and outcome measures across the included publications, findings were organized by prehabilitation component (physical exercise, nutritional support, psychological support, and multimodal approaches) and by outcome domain, and were qualitatively interpreted rather than statistically pooled. As this article was not conducted as a systematic review or umbrella review, formal procedures such as protocol registration, duplicate screening, overlap analysis, AMSTAR-2 assessment, ROBIS evaluation, or GRADE-based certainty appraisal were not performed.

## 3. Results

Beyond the well-established roles of exercise, nutritional optimization, and psychological support, contemporary prehabilitation increasingly encompasses patient-centered interventions such as stoma education, sexual health counselling, and shared decision-making, which are discussed later in this review. Across the available literature, improvements in functional outcomes (e.g., functional capacity and cardiorespiratory fitness) were reported more frequently than improvements in major clinical endpoints such as postoperative complications or length of hospital stay. Accordingly, these outcome domains are discussed separately below.

### 3.1. Physical Exercise

The synthesis of the current literature demonstrates that preoperative physical conditioning was reported to improve patients’ functional capacity, as evidenced by significant improvements in the 6-min walk test (6 MWT) distance and peak oxygen uptake (VO_2_peak) [14,15,16,17]. However, this robust physiological optimization does not uniformly translate into hard clinical outcomes. While several reviews have reported that targeted interventions—such as inspiratory muscle training (IMT)—significantly mitigate postoperative pulmonary complications [18,19], the impact of general aerobic exercise on overall complication rates and total length of hospital stay (LOS) remains mixed and highly dependent on patient adherence and baseline risk profiles [14,20,21,22].

### 3.2. Nutritional Support

When evaluating nutritional interventions, the narrative evidence reveals distinct variations in efficacy based on the specific modality used. Reductions in postoperative infectious complications and shorter hospital stays were most frequently reported for immunonutrition protocols (enriched with arginine, omega-3 fatty acids, and nucleotides) administered to patients undergoing major colorectal resections [18,23]. In contrast, the effects of standard Oral Nutritional Supplements (ONS) were associated with clinical benefit primarily in cohorts presenting with baseline malnutrition [15,24]. Isolated dietary counseling, without direct supplementation, was the least frequently studied nutritional strategy, and the available literature does not converge on a clear impact on objective surgical outcomes [14,25].

Across the included reviews, reported nutritional protocols varied considerably in energy and protein targets, timing of initiation relative to surgery, treatment duration, and the degree to which supplementation was individualized to patients’ baseline nutritional status. Oral nutritional supplementation was typically initiated in the pre-operative period and, in some protocols, continued postoperatively; however, specific energy and protein targets were inconsistently reported and were rarely individualized according to body weight, estimated requirements, or baseline nutritional risk. Adherence to supplementation appeared higher in supervised or hospital-based protocols than in unsupervised home-based regimens, although formal adherence data were not consistently provided across studies.

In addition, immunonutrition was frequently treated as a single category in the included reviews despite substantial differences in formula composition. Reported products contained arginine, omega-3 fatty acids, and nucleotides in varying combinations and concentrations, were administered over differing perioperative windows and durations, and were applied across heterogeneous surgical contexts (e.g., open versus minimally invasive resections) and baseline nutritional profiles. This heterogeneity in formula composition, perioperative timing, duration, surgical context, and baseline nutritional status limits direct comparability across studies and should be considered when interpreting the narrative conclusions regarding the clinical benefit of immunonutrition.

### 3.3. Psychological Support

Psychological interventions within prehabilitation primarily affect patient-reported outcome measures rather than surgical parameters. Multiple reviews reported reductions in preoperative anxiety and depression scores (e.g., via HADS scales) and improvements in early HRQoL [14,26]. However, direct correlations between psychological conditioning alone and reductions in LOS or physical complications are weak [20,25]. As an extension of this psychosocial preparation, the literature increasingly underscores the necessity of addressing specific, high-impact functional disruptions. Preoperative stoma education focusing on psychophysical adaptation, as well as stoma site marking alongside proactive counseling regarding sexual health and intimacy, are identified as critical yet historically marginalized components that significantly determine long-term social readaptation and post-surgical well-being [27].

### 3.4. Multimodal Prehabilitation

The synthesis of multimodal pathways (combining physical, nutritional, and psychological elements) highlights a synergistic effect that exceeds the impact of isolated unimodal interventions, particularly in accelerating the trajectory of post-operative functional recovery back to baseline [14,24,28]. Multimodal prehabilitation has been associated with reductions in postoperative complications in several contemporary systematic reviews and meta-analyses, particularly when implemented within established perioperative care pathways such as ERAS [22,25]. However, these findings should be interpreted with caution given the substantial heterogeneity of study populations, intervention protocols, outcome measures, and baseline perioperative care across the included studies [20,26].

Reviews incorporating real-world data further suggest that the effectiveness of multimodal prehabilitation may be underestimated in RCTs, in which patient selection and a high standard of usual care limit the ability to demonstrate an additional effect of the intervention [24]. At the same time, it should be emphasized that heterogeneity of multimodal protocols, variable intervention duration, lack of standardization of training intensity, and variable patient adherence significantly hinder comparison of results across studies [14,19,29]. Studies assessing the therapeutic quality of interventions indicate that the absence of clinical effects in some trials more often results from insufficient quality of prehabilitation program design rather than from ineffectiveness of the multimodal concept itself [30]. Overall, the available evidence suggests that the effectiveness of multimodal prehabilitation is determined both by appropriate patient selection and by the quality, intensity, and supervision of intervention delivery [30,31].

Characteristics and principal findings of the review-level publications informing this narrative synthesis are presented in Table 1, organized by prehabilitation component, population, intervention, and reported outcomes.

### 3.5. Psychosocial and Specific Functional Interventions

Beyond the core pillars of nutrition and physical exercise, a truly comprehensive, patient-centered prehabilitation model must also proactively address specific, high-impact functional challenges often neglected in early care, such as stoma education and sexual health counseling. Furthermore, maximizing post-surgical quality of life requires that prehabilitation programs integrate tailored supportive interventions well before the operation. Specifically, pre-operative stoma education including marking the optimal stoma site and psychological bracing and early counseling on sexual health are critical to mitigating the profound psychosocial and physical disruptions associated with colorectal resections. Rather than peripheral additions, these components represent logical extensions of psychological and functional conditioning.

Future systematic reviews and meta-analyses addressing prehabilitation in the surgical treatment of CRC should more extensively incorporate aspects of patient preparation for potential stoma formation, including preoperative education, psychophysical adaptation, and procedures for selection and fitting of stoma equipment. These elements are of significant importance for postoperative functioning, quality of life, and the process of social readaptation, yet they remain marginalized in current analyses of prehabilitation effectiveness. At the same time, an important and insufficiently explored area is educational and psychological support related to sexual health, which may influence both the psychophysical well-being of patients and long-term outcomes of surgical treatment [27]. Inclusion of these components in future analyses would allow for a more holistic evaluation of prehabilitation as an intervention preparing the patient not only for surgery, but also for life after oncological treatment.

### 3.6. Patient Tolerance and Safety

Across all included systematic reviews and meta-analyses, preoperative prehabilitation was repeatedly reported as a safe and well-tolerated intervention in patients with CRC, regardless of the scope of applied components [14,20,29]. Both unimodal and multimodal programs were not associated with an increased incidence of adverse events, deterioration of patients’ clinical condition, or delays in surgical treatment [16,25,33]. Reports of intervention discontinuation most commonly referred to organizational factors, low adherence, or deterioration of general condition related to the underlying malignancy, rather than to direct complications of prehabilitation [21]. Of particular importance is the observation that prehabilitation was also well tolerated in older patients and in those burdened with comorbidities, provided that appropriate individualization of the program and specialist supervision were ensured [17,31].

Multimodal programs delivered in supervised or hybrid models were characterized by higher adherence and lower discontinuation rates compared with exclusively home-based interventions [22,31]. None of the reviewed studies demonstrated an increased risk of severe complications (Clavien–Dindo ≥ III) associated with participation in prehabilitation [15,22]. The available data regarding the safety of nutritional and psychological components likewise do not indicate significant risks [23,28]; however, authors of several studies emphasize the need for cautious and meticulous patient selection for immunonutrition supplementation and for monitoring potential interactions with oncological treatment [27]. It is evident that prehabilitation, particularly in a multimodal model, can be regarded as a safe strategy supporting surgical treatment of CRC. In summary, the potential benefit profile for the patient outweighs the minimal risks associated with the implementation of prehabilitation.

## 4. Discussion

### 4.1. Main Conclusions from the Current State of Knowledge

The current body of evidence suggests that prehabilitation represents a promising perioperative strategy for improving physiological reserve and supporting postoperative recovery in patients undergoing CRC surgery. Findings were most concordant for improvements in functional outcomes, particularly functional capacity and cardiorespiratory fitness, whereas evidence regarding hard clinical endpoints, including postoperative complications, length of hospital stay, readmissions, and mortality, varied more across studies owing to substantial heterogeneity in intervention protocols, patient populations, and outcome assessment [14,15,16,17,18,19,20,21,22,23,24,25,26,27,28,29,30,31,32,33]. Accordingly, improvements in functional performance should not be interpreted as directly equivalent to improvements in major clinical outcomes.

Overall, multimodal prehabilitation integrating exercise, nutritional optimization, and psychological support was the approach most frequently associated with favorable outcomes across the included publications. Compared with unimodal interventions, multimodal programmes appear to provide greater improvements in functional recovery and have been associated with reductions in postoperative complication rates and hospital length of stay in several contemporary systematic reviews and meta-analyses [22,25]. Nevertheless, these findings should be interpreted cautiously because of considerable methodological heterogeneity and differences in baseline perioperative care across the available literature [20,26].

Nutritional optimization appears to represent one of the key components of multimodal prehabilitation. In addition to improving nutritional status, nutritional interventions—particularly oral nutritional supplementation and immunonutrition—have been associated with reductions in infectious complications and improved postoperative recovery, especially among malnourished or metabolically compromised patients [18,23]. Furthermore, adequate nutritional support provides the physiological basis for effective adaptation to exercise training, highlighting the complementary relationship between nutritional and exercise interventions within multimodal programmes.

Although oral nutritional supplementation and immunonutrition appear beneficial, the biological interactions between specific immunonutrients (e.g., arginine and omega-3 fatty acids) and tumor immunity remain incompletely understood. While current evidence does not indicate clinically relevant oncological safety concerns, further translational studies are needed to clarify the underlying mechanisms and identify patient subgroups most likely to benefit from individualized immunonutritional strategies.

An important finding emerging from the available literature is the discrepancy between randomized controlled trials and real-world observational studies. These differences may be explained by stricter patient selection, higher baseline quality of perioperative care, and greater protocol standardization in randomized trials, whereas observational studies more closely reflect routine clinical practice and often include higher-risk patient populations. In addition, contemporary “usual care” groups frequently receive several components of Enhanced Recovery After Surgery (ERAS) pathways, including nutritional assessment, carbohydrate loading, smoking cessation counselling, anaemia correction, and early mobilization. Consequently, the incremental benefit attributable exclusively to structured prehabilitation may be attenuated in centres with high ERAS compliance. Differences in intervention intensity, programme duration, patient adherence, and therapeutic quality may further contribute to the variability of reported clinical outcomes [24,30].

Across the available literature, prehabilitation is repeatedly reported as a safe and well-tolerated intervention when appropriately individualized and delivered under adequate supervision. No convincing evidence indicates an increased risk of intervention-related adverse events, supporting its role as a complementary component of contemporary perioperative care [14,15,16,17,18,19,20,21,22,23,24,25,26,27,28,29,30,31,32,33].

From a clinical perspective, the beneficial effects of prehabilitation are most plausibly explained by preservation of skeletal muscle mass and function, improvement of nutritional and metabolic status, attenuation of systemic inflammation, maintenance of intestinal barrier integrity, and modulation of psychological stress responses. Together, these mechanisms enhance physiological resilience and improve patients’ ability to tolerate surgical stress, providing a clinically meaningful explanation for the functional benefits reported across the literature [18,20,23,27].

Recently, a narrative review synthesizing evidence from primary clinical studies has also confirmed the favorable effects of multimodal prehabilitation on functional outcomes while emphasizing the continued variability regarding major postoperative endpoints, further highlighting the need for evidence integration at different levels of synthesis [34].

### 4.2. Indications for Future Research Directions and Implementation Work

Despite the growing body of evidence supporting prehabilitation in CRC surgery, several important knowledge gaps remain. Future research should move beyond establishing whether prehabilitation is beneficial and instead focus on identifying which patients benefit most, which intervention components are essential, and how prehabilitation can be implemented most effectively in routine clinical practice.

A major research priority is the standardization of multimodal prehabilitation programmes. Future randomized controlled trials should incorporate clearly defined intervention protocols, including standardized exercise prescription, nutritional optimization, psychological support, programme duration, supervision, adherence monitoring, and reporting of intervention fidelity. Greater standardization of study design would substantially improve comparability across studies and facilitate translation into clinical practice.

Further studies should also emphasize patient stratification to identify populations most likely to benefit from prehabilitation. Particular attention should be given to older adults, patients with frailty, sarcopenia, malnutrition, multimorbidity, or reduced functional capacity. The incorporation of standardized screening tools assessing functional status, nutritional risk, frailty, and psychological burden may support more personalized and resource-efficient perioperative care.

Another important direction is the integration of clinical outcomes with biological and translational research. Simultaneous evaluation of inflammatory, metabolic, immunological, and nutritional biomarkers may improve understanding of the mechanisms underlying prehabilitation and facilitate the identification of predictors of treatment response.

Finally, implementation-oriented research should evaluate the feasibility, acceptability, cost-effectiveness, and scalability of prehabilitation programmes under real-world healthcare conditions. Comparative studies assessing supervised, hybrid, and home-based models, together with analyses of their integration within established Enhanced Recovery After Surgery (ERAS) pathways, are essential for developing sustainable and widely applicable models of perioperative care.

### 4.3. Practical Recommendations for Health Care Systems

Based on the current state of knowledge, several practical recommendations can be formulated to support the implementation of prehabilitation in patients undergoing CRC surgery. Prehabilitation should be regarded not as an optional intervention but as an integral component of comprehensive perioperative care, complementing established ERAS pathways.

Successful implementation requires consideration of several organizational domains. Feasibility depends on the availability of trained multidisciplinary personnel (e.g., physiotherapists, dietitians, nurses, and clinical psychologists), infrastructure for supervised or hybrid programme delivery, and integration within existing perioperative care pathways. Adoption is influenced by institutional priorities, clinician engagement, and patient acceptance, whereas long-term sustainability requires standardized intervention protocols, monitoring of adherence, and adequate organizational resources.

Priority should be given to patients at increased perioperative risk, including older adults and those presenting with frailty, sarcopenia, malnutrition, multimorbidity, or reduced functional capacity. Patient stratification should be supported by standardized screening tools, including the Clinical Frailty Scale (CFS) for frailty assessment, Nutritional Risk Screening 2002 (NRS-2002) or the Patient-Generated Subjective Global Assessment (PG-SGA) for nutritional risk, and functional performance measures such as the 6-min walk test (6 MWT). Assessment of psychological burden should also be considered where appropriate. Early implementation of these assessments during surgical qualification may facilitate timely referral to individualized prehabilitation programmes.

Current evidence supports implementation of multimodal prehabilitation programmes combining structured exercise, nutritional optimization, and psychological support. Effective programme delivery requires individualized exercise prescription, appropriate therapeutic intensity, regular monitoring of adherence, and periodic assessment of patient progress. Standardized intervention protocols may improve reproducibility and facilitate implementation across different healthcare settings. From an organizational perspective, prehabilitation should be integrated within existing perioperative care pathways rather than introduced as a parallel service. Close collaboration between surgeons, anaesthesiologists, nurses, physiotherapists, dietitians, and clinical psychologists is essential to ensure coordinated patient management and continuity of care throughout the perioperative period.

Patient education should also include preparation for potential stoma formation, realistic expectations regarding postoperative recovery, and counselling addressing body image, intimacy, and sexual health whenever clinically appropriate. These patient-centred interventions may facilitate psychological adaptation, improve treatment acceptance, and support postoperative quality of life.

In practical terms, implementation may follow a stepwise pathway consisting of: (1) early identification of eligible patients during surgical qualification; (2) risk stratification based on functional capacity, nutritional status, frailty, and psychological burden; (3) initiation of an individualized multimodal prehabilitation programme; (4) monitoring of adherence and adjustment of intervention intensity according to patient progress; and (5) integration of prehabilitation within established ERAS pathways supported by interdisciplinary coordination and continuous outcome evaluation.

Implementation outcomes should be evaluated using predefined indicators, including programme uptake, adherence, completion rate, postoperative complications, length of hospital stay, patient-reported outcomes, and cost-effectiveness, to support continuous quality improvement and facilitate comparison across healthcare settings.

### 4.4. Limitations

There are several limitations that should be acknowledged. Due to the narrative design of the present review, no formal methodological quality appraisal of the included systematic reviews was performed using standardized tools such as AMSTAR-2 or ROBIS. Consequently, the methodological quality of the included reviews was not formally compared, which may introduce a degree of selection bias. However, this approach is consistent with the narrative design of the review, whose objective was to provide an interpretative clinical synthesis rather than a formal quantitative overview of reviews. The focus on contemporary, peer-reviewed review-level evidence was intended to mitigate this limitation.

The literature search was limited to the PubMed database and did not include additional databases (e.g., Embase, Cochrane Library) or gray literature sources, which may have resulted in omission of some relevant publications. However, PubMed was selected as the primary biomedical database due to its broad coverage of peer-reviewed medical literature, including the majority of relevant systematic reviews and meta-analyses in this field.

In addition, potential overlap of primary studies across included systematic reviews and meta-analyses was not formally assessed, which may lead to partial duplication of evidence and overrepresentation of certain findings. Given this, the findings should be interpreted as a qualitative synthesis of evidence trends rather than as independent confirmations of effect sizes.

## 5. Conclusions

The current body of evidence indicates that prehabilitation is a safe and clinically promising adjunct to perioperative care for patients undergoing CRC surgery. The most frequently reported benefits have been demonstrated for improvements in functional capacity, whereas evidence regarding postoperative complications, length of hospital stay, and other major clinical outcomes varies more across studies.

Among the available approaches, multimodal prehabilitation integrating structured exercise, nutritional optimization, and psychological support appears to provide the greatest potential to enhance perioperative resilience and support postoperative recovery. The effectiveness of these programmes is likely influenced by intervention quality, appropriate patient selection, and integration within established perioperative care pathways.

Overall, contemporary evidence supports the incorporation of multimodal prehabilitation into routine perioperative management of patients undergoing CRC surgery, particularly in individuals at increased perioperative risk. Continued standardization of intervention protocols and high-quality clinical research will further strengthen the evidence base and facilitate broader implementation in clinical practice.

## Figures and Tables

**Table 1:** Characteristics and principal findings of the review-level publications included in the narrative synthesis.

Authors, Year, Journal	Type of Review	Number of Studies and Patients	Population Focus	Prehabilitation Model	Main Indicators	Key Findings	Implications for Prehabilitation	Prehabilitation Safety
1. Hijazi et al., 2017; Int. J. Surg. [14]	Systematic review (PRISMA)	9 prospective comparative studies (7 RCTs, 2 prospective non-RCTs); N = 549 patients (281 prehabilitation vs. 268 control); 5 studies included CRC surgery	Predominantly CRC surgery	Predominantly exercise-based prehabilitation; two studies implemented trimodal programs including physical exercise, nutritional support, and psychological intervention	Functional capacity (6 MWT), cardiopulmonary fitness (VO_2_max, anaerobic threshold), psychological status (HADS), health-related quality of life (SF-36), postoperative complications (Clavien–Dindo)	Prehabilitation improved functional capacity in several studies (increased 6 MWT distance and cardiopulmonary fitness parameters); however, reductions in postoperative complications or length of hospital stay were not demonstrated	Evidence suggests potential benefits of prehabilitation in improving preoperative functional reserve; however, substantial heterogeneity in intervention design, duration (2–8 weeks), delivery, and outcome measures highlights the need for standardized protocols and larger trials	No safety concerns were reported across included studies; prehabilitation programs were considered feasible and generally safe
2. Falz et al., 2022; J. Cancer Res. Clin. Oncol. [15]	Systematic review and meta-analysis	23 studies included in systematic review; 14 controlled trials in meta-analysis; 1648 total patients (approx. 719 prehabilitation, 742 control)	CRC surgery	Exercise-based prehabilitation (aerobic training ± resistance training ± inspiratory muscle training); in several trials multimodal approaches including nutrition and psychological support	Functional capacity (6 MWD; VO_2_max); postoperative complications (Clavien–Dindo/CCI); length of hospital stay (LOS)	Significant improvement in functional capacity (6 MWD mean difference ≈ 30.8 m; 95% CI 13.3–48.3; *p* = 0.0005). No significant reduction in overall postoperative complications (OR 0.84) or LOS (MD −0.27 days). Prehabilitation lasting >3 weeks showed a trend toward lower complication rates	Exercise-based prehabilitation improves preoperative functional capacity in CRC patients; programs lasting >3 weeks may provide greater clinical benefit. However, heterogeneity of protocols and outcomes limits translation into standardized clinical practice	Exercise programs demonstrated high adherence (≈68–98%) and very low incidence of adverse events; no major safety concerns reported
3. Molenaar CJL et al., 2022; Cochrane Database of Systematic Reviews [32]	Systematic review and meta-analysis (Cochrane)	3 RCTs; 250 patients (130 prehabilitation, 120 control)	CRC surgery	Multimodal prehabilitation programmes including moderate-intensity exercise, nutritional support, and psychological intervention implemented approximately 4 weeks before surgery	Functional capacity (6 MWT); postoperative complications (within 30 days); HRQoL (SF-36, HADS); length of hospital stay; emergency department visits; readmissions	Prehabilitation improved presurgical functional capacity (MD ≈ 24.9 m in 6 MWT; 95% CI 11.24–38.57). Postoperative functional capacity showed a non-significant trend favoring prehabilitation at 4 weeks (MD ≈ 26.0 m) and 8 weeks (MD ≈ 26.6 m). No significant difference in postoperative complications (RR ≈ 0.95) or length of hospital stay. Evidence certainty ranged from moderate to very low	Multimodal prehabilitation may improve preoperative functional capacity and potentially support postoperative recovery; however, evidence remains limited due to small number of RCTs and methodological heterogeneity	Safety outcomes insufficiently reported; available trials did not indicate major safety concerns or increased complication risk related to prehabilitation
4. Bausys et al., 2022; Cancers [20]	Comprehensive narrative review	20 clinical studies (10 RCTs, 9 pilot studies, 1 retrospective cohort study); sample sizes across studies ranged from 9 to 351 CRC patients	CRC surgery	Both unimodal (exercise-based) and multimodal programs (exercise + nutritional support + psychological interventions) implemented mainly 2–6 weeks before surgery or during neoadjuvant therapy	Physical fitness parameters (VO_2_peak, VO_2_ at ventilatory anaerobic threshold, 6 MWT), muscle strength, lean body mass, postoperative complications, length of hospital stay, QoL, adherence	Most studies reported improvements in physical capacity (VO_2_peak, 6 MWT), muscle strength, and nutritional status. Some studies showed reduced postoperative complications and shorter hospital stay; however, findings varied across trials	Prehabilitation may improve patients’ functional reserve before surgery and support recovery, but optimal protocols (unimodal vs. multimodal, duration, supervision) remain unclear due to heterogeneity of current evidence	Exercise-based and multimodal prehabilitation programs were generally reported as safe and feasible, with high adherence rates and no major adverse events reported in most studies
5. Franssen et al., 2024; Eur J Surg Oncol. [24]	Systematic review with meta-analyses (RCTs vs. OCSs)	Observational studies and RCTs evaluating prehabilitation before CRC surgery	CRC surgery	Prehabilitation programs including exercise-based or multimodal interventions implemented before CRC surgery	Postoperative complications; length of hospital stay (LoS); patient characteristics (age, ASA score); intervention characteristics	Meta-analysis showed significant reductions in postoperative complications (OR 0.54; 95% CI 0.40–0.72) and LoS (MD −1.34 days; 95% CI −2.57 to −0.12) in observational studies, whereas RCTs did not demonstrate significant differences (complications OR 0.95; LoS MD 0.16)	Real-world observational data suggest that prehabilitation may improve postoperative outcomes in CRC surgery, particularly regarding complication rates and hospital stay, highlighting the importance of evaluating effectiveness in real-life clinical settings	Safety outcomes were not reported as a major concern; included studies generally indicated feasibility of prehabilitation programs in clinical practice
6. Gennuso et al., 2024; BMC Cancer [33]	Systematic review and meta-analysis (RCTs only)	42 RCTs included in systematic review; 13 RCTs included in meta-analysis; mixed cancer populations (lung ≈ 38%, CRC ≈ 27%, prostate ≈ 18%) approx. 27%	Mixed cancer populations (CRC subgroup)	Predominantly exercise-based prehabilitation (aerobic and resistance training, respiratory exercises, HIIT, pelvic floor training); occasionally combined with breathing or functional training	Functional capacity (6 MWT), anxiety and depression (HADS), postoperative complications (Clavien–Dindo), quality of life (SF-36, FACT), physical performance tests	Meta-analysis demonstrated significant improvement in functional capacity (6 MWT MD ≈ +38.53 m; 95% CI 33.03–44.04). Significant reductions in anxiety (HADS-A MD −0.49) and depression (HADS-D MD −0.71). No significant differences in postoperative complications	Prehabilitation appears effective in improving functional capacity and psychological outcomes before cancer treatment; however, heterogeneity of interventions and tumor types limits disease-specific conclusions (including CRC)	No significant safety concerns reported across included trials; exercise-based prehabilitation interventions were generally feasible and well tolerated
7. Marmol-Perez et al., 2024; Dis Colon Rectum [26]	Systematic review and meta-analysis	20 studies; N = 3805 patients (12 RCTs, 5 non-RCTs, 3 before-after studies)	CRC surgery	Multidisciplinary (multimodal) prehabilitation programs including exercise-based training combined with nutritional and supportive interventions before CRC surgery	Functional capacity (VO_2_peak, 6 MWT), postoperative hospital length of stay	Meta-analysis demonstrated significant reductions in postoperative hospital length of stay (≈−2 days) and improvements in functional capacity (VO_2_peak effect size d ≈ 0.27; 6 MWT d ≈ 0.31)	Multidisciplinary prehabilitation may improve physiological reserve and functional capacity before CRC surgery and may contribute to shorter hospitalization	No major safety concerns were reported; interventions were generally well tolerated across studies
8. Steffens et al., 2024; Ann Surg Oncol. [18]	Systematic review and meta-analysis (RCTs only; PRISMA, GRADE)	23 RCTs; N = 2475 patients undergoing CRC surgery	CRC surgery	Exercise-based, nutrition-based, psychological, and multimodal prehabilitation interventions	Postoperative complications (overall and infectious), length of hospital stay	Nutritional prehabilitation reduced infectious complications (RR ≈ 0.65) and shortened hospital stay (≈−0.87 days). Exercise, psychological, and multimodal interventions showed variable effects on postoperative outcomes	Nutritional prehabilitation has the strongest evidence for improving postoperative outcomes; the effectiveness of other modalities remains uncertain and requires further high-quality RCTs	No significant safety concerns reported; interventions were generally well tolerated
9. Wee et al., 2024; Ann Coloproctol. [29]	Systematic review and meta-analysis (RCTs and non-RCTs; PRISMA)	7 studies; N = 1042 CRC patients (PH: 382, control: 660)	CRC surgery	Trimodal prehabilitation including exercise training, nutritional optimization, and psychological support	Postoperative complications (Clavien–Dindo I–II and ≥III), length of hospital stay, operative time, 30-day readmission; secondary outcomes: 6 MWT, HADS	Prehabilitation showed no significant reduction in postoperative complications, length of stay, operative time, or readmission. Some studies reported improved postoperative functional capacity (6 MWT), but findings varied across studies	Current evidence does not confirm clear short-term clinical benefits of trimodal prehabilitation before CRC surgery; further large RCTs are needed to determine long-term and functional outcomes	Prehabilitation programs were feasible and generally safe, with no major adverse events reported
10. Zhang et al., 2024; Integr Cancer Ther. [25]	Systematic review and meta-analysis of RCTs (PRISMA)	Systematic review and meta-analysis of RCTs (PRISMA; PROSPERO	CRC surgery	Preoperative lifestyle interventions and prehabilitation including exercise training, nutritional support, psychological support, or multimodal combinations	(6 MWT), postoperative complications, QoL, total and postoperative length of hospital stay (tLHS, postLHS), healthcare expenditure	Prehabilitation significantly improved postoperative functional capacity (6 MWT) and reduced complication rates. No significant improvements were observed in QoL or hospital length of stay. Considerable heterogeneity was present across studies	Preoperative lifestyle management and multimodal prehabilitation may enhance postoperative functional recovery and reduce complications in CRC surgery, but standardized protocols and higher-quality trials are required	Interventions were generally feasible and safe, with no major safety concerns reported in the included RCTs
11. Gao et al., 2025; Front Oncol. [19]	Scoping review (PRISMA-ScR framework)	12 studies (9 RCTs, 1 cohort study, 1 pilot study, 1 emulated target trial); several hundred patients	CRC surgery	12 studies (9 RCTs, 1 cohort study, 1 pilot intervention study, 1 emulated target trial); several hundred CRC patients	12 studies (9 RCTs, 1 cohort study, 1 pilot intervention study, 1 emulated target trial); several hundred CRC patients	Functional capacity (6 MWT), cardiopulmonary fitness, nutritional status (e.g., PG-SGA), psychological outcomes (HADS, GAD-7, PHQ-9), postoperative complications (Clavien–Dindo), length of hospital stay, HRQoL	Multimodal prehabilitation demonstrated beneficial trends in improving functional capacity and overall clinical prognosis in patients undergoing CRC surgery; however, substantial heterogeneity in program content, duration (2–8 weeks), and outcome measures limited quantitative synthesis	Multimodal prehabilitation appears promising for improving perioperative functional reserve and supporting postoperative recovery; further high-quality trials are needed to standardize protocols and evaluation metrics
12. Grońska et al., 2025; Pol Arch Intern Med. [23]	Systematic review (PRISMA 2020)	12 studies (11 RCTs, 1 observational study); gastrointestinal cancers including colorectal, gastric, and pancreatic cancer	Mixed gastrointestinal cancers	Nutrition-based prehabilitation focusing on oral nutritional supplements (ONS) and immunonutrition before oncological treatment	Postoperative complications (including infectious complications), nutritional status (e.g., PG-SGA, albumin, prealbumin), body weight, length of hospital stay, QoL	Dietary prehabilitation, particularly ONS and immunonutrition, was associated with improved nutritional status and reduced postoperative complications, especially in malnourished patients. Findings for reductions in length of hospital stay and quality-of-life improvements varied due to heterogeneity across studies	Nutritional prehabilitation should be considered an important component of oncological prehabilitation, particularly in patients at risk of malnutrition; integration with multimodal programs and individualized nutritional strategies is recommended	Interventions were generally safe and well tolerated; no significant safety concerns or intervention-related adverse events were reported
13. Hirst et al., 2025; Support Care Cancer [28]	Systematic review and meta-analysis of RCTs (PRISMA; GRADE)	18 RCTs included in systematic review; 11 RCTs in meta-analysis; N = 1612 patients (meta-analysis N = 923) undergoing cancer surgery	Mixed oncological surgery	Psychological prehabilitation, implemented as a unimodal intervention or as part of multimodal programs (exercise, nutrition, psychological support)	Postoperative complications, length of hospital stay	Psychological prehabilitation significantly reduced overall postoperative complications (RR ≈ 0.73; 95% CI 0.60–0.89), with similar effects observed in abdominal cancer surgery (RR ≈ 0.65). No statistically significant reduction in length of hospital stay was observed	Psychological interventions represent an important component of multimodal prehabilitation programs and may improve perioperative outcomes in oncological surgery	Psychological interventions represent an important component of multimodal prehabilitation programs and may improve perioperative outcomes in oncological surgery
14. Jetten et al., 2025; Colorectal Dis. [30]	Systematic review and meta-analysis of RCTs (PRISMA; Cochrane)	Systematic review and meta-analysis of RCTs (PRISMA; Cochrane methodology; PROSPERO registered)	CRC surgery	Exercise-based prehabilitation (unimodal or multimodal), typically including aerobic training ± resistance training and sometimes nutritional or psychological components	Preoperative aerobic fitness (e.g., 6 MWT, VO_2_peak), postoperative complications (overall and severe), length of hospital stay (LOS)	Prehabilitation significantly improved preoperative aerobic fitness (e.g., increased 6 MWT distance), but pooled analyses did not show significant reductions in overall postoperative complications, severe complications, or LOS. Only one study demonstrated both low risk of bias and high therapeutic quality of the intervention	Results highlight the importance of improving the therapeutic quality and reporting of exercise-based prehabilitation programmes (e.g., adequate training dosage, supervision, adherence). Future trials should incorporate structured tools such as i-CONTENT during intervention design	Prehabilitation programmes were generally safe and feasible, with very few reported adverse events across studies.
15. Li et al., 2025; Front Med. [16]	Systematic review and network meta-analysis of RCTs (PRISMA	27 RCTs; N = 2946 patients undergoing CRC surgery	CRC surgery	Four types of prehabilitation compared: breath-only training (BT), exercise-only (EX), nutrition-only (NU), and multimodal prehabilitation (exercise + nutritional + psychological interventions)	Postoperative complications, length of hospital stay, 6 MWT, anxiety and depressive symptoms	Multimodal prehabilitation significantly reduced postoperative complications (OR ≈ 0.47) and length of hospital stay (MD ≈ −1.17 days), improved preoperative functional capacity (6 MWT), and reduced preoperative anxiety. Other single-modality strategies showed variable effects	Findings support multimodal prehabilitation as the most effective strategy for optimizing surgical outcomes in CRC patients, highlighting the importance of integrated exercise, nutritional, and psychological preparation before surgery	Interventions were generally safe; no major safety concerns were reported across the included RCTs
16. Liao et al., 2025; J Am Geriatr Soc. [31]	Systematic review and meta-analysis (PRISMA)	14 studies (7 RCTs, 5 prospective cohort, 2 retrospective cohort); N = 2314 patients undergoing CRC surgery	CRC surgery	Multimodal prehabilitation including exercise training, nutritional optimization, and psychological support; in some studies extended with geriatric consultation or cardiovascular risk optimization; interventions typically implemented 3–6 weeks before surgery	Length of hospital stay, postoperative complications, time to first passage of flatus, 30-day readmission rate, emergency department visits; functional capacity assessed mainly by 6 MWT	Multimodal prehabilitation significantly reduced length of hospital stay (MD ≈ −2.47 days), postoperative complication rate (OR ≈ 0.74), and accelerated bowel recovery (earlier first flatus). Supervised exercise produced stronger effects than unsupervised programs	Evidence supports multimodal prehabilitation as an effective strategy to improve postoperative recovery in CRC surgery, particularly in older and high-risk patients; supervised exercise and nutritional optimization appear key components	Interventions were generally safe; no major safety concerns were reported, although some included studies showed moderate risk of bias and potential publication bias for length-of-stay outcomes
17. Machado et al., 2024; Supportive Care in Cancer [21]	Systematic review and meta-analysis (RCTs only; PRISMA, RoB2, GRADE)	8 RCTs; N = 1092 patients undergoing CRC surgery (533 prehabilitation vs. 559 control)	CRC surgery	Home-based exercise prehabilitation, predominantly multimodal (exercise training ± nutritional and psychological interventions); aerobic + resistance training most common; 2–4 weeks before surgery; some studies continued rehabilitation postoperatively	Postoperative exercise capacity (6 MWT), postoperative complications (Clavien–Dindo classification), length of hospital stay, health-related quality of life (HRQoL; SF-36)	No significant effect of home-based exercise prehabilitation on postoperative complications (RR ≈ 1.00) or length of hospital stay (MD ≈ −0.20 days). Primary analysis suggested improvement in 6 MWT distance (≈30 m), but sensitivity analysis including only high-quality trials showed no significant effect. HRQoL outcomes were inconclusive due to limited evidence	Current evidence does not support home-based exercise prehabilitation as an effective strategy to improve surgical outcomes in CRC. Low training intensity, short intervention duration, and variable adherence may limit effectiveness. Higher-intensity or supervised programs may be required	Interventions were generally safe; few adverse events reported (e.g., musculoskeletal discomfort or dizziness in isolated cases). No major safety concerns across included trials
18. Maroto-Izquierdo et al., 2025; Healthcare [17]	Systematic review and meta-analysis (RCTs only; PRISMA, RoB2, GRADE)	6 RCTs; N = 379 CRC patients scheduled for surgery (192 prehabilitation vs. 187 control)	CRC surgery	Exercise-based prehabilitation using concurrent training (combined aerobic + resistance exercise), typically 2–6 weeks before surgery, 2–4 sessions/week (most commonly 3 sessions/week); sessions ~50–60 min	Functional capacity measured primarily with the 6 MWT; additional measures included gait speed, lower limb strength, inspiratory muscle strength, and functional mobility tests	Improvement in functional capacity in the exercise group compared with standard care (SMD = 0.28; 95% CI 0.03–0.54). Greater benefits in patients <70 years (SMD ≈ 0.48) compared with ≥70 years (SMD ≈ 0.10). Meta-regression found no significant association between outcomes and BMI, baseline walking capacity, or intervention duration	Concurrent aerobic-resistance exercise programs implemented 2–4 weeks before surgery may improve preoperative functional capacity and potentially reduce perioperative risk in CRC patients; individualized protocols are recommended to maximize effectiveness	Exercise-based prehabilitation showed low risk of adverse events and was considered safe; supervised programs may improve adherence and reduce risk of complications during training
19. Widanage et al., 2025; Cureus [22]	Systematic review and meta-analysis (RCTs only; PRISMA, RoB2, GRADE)	5 RCTs; N = 422 patients undergoing elective CRC surgery	CRC surgery	Multimodal prehabilitation programs combining ≥2 components: structured exercise training, nutritional support, psychological interventions; typically delivered for ~4 weeks preoperatively, either home-based or supervised within hospital settings	Postoperative complications (overall), severe complications (Clavien–Dindo ≥III or CCI > 20), and functional capacity measured by the 6 MWT	Significant reduction in overall postoperative complications with multimodal prehabilitation (OR = 0.60; 95% CI 0.42–0.86; I^2^ = 0%). Functional recovery improved significantly with an average increase of ~50.8 m in 6 MWT distance at 8 weeks postoperatively. Reduction in severe complications was observed but not statistically significant (OR = 0.65; 95% CI 0.13–3.29)	Multimodal prehabilitation may enhance perioperative resilience, improve postoperative functional recovery, and reduce morbidity when integrated with ERAS pathways. Programs combining exercise, nutrition, and psychological support appear most effective, particularly when implemented ~4 weeks before surgery	Interventions were generally feasible and safe, with no evidence of harm across trials; adherence to multimodal programs was high, suggesting practical implementation within perioperative care pathways
20. Włodarczyk, 2025; Int J Mol Sci. [27]	Narrative review (clinical and molecular synthesis)	Systematic reviews/meta-analyses and clinical studies (no original quantitative synthesis)	CRC surgery (clinical synthesis)	Evidence synthesized from clinical studies, systematic reviews, and mechanistic studies on CRC prehabilitation (no original quantitative meta-analysis)	Multimodal prehabilitation including nutritional optimization, structured physical exercise, psychological interventions, with additional discussion of molecular pathways (inflammation, immune activation, metabolic regulation)	Multimodal prehabilitation is associated with improved functional capacity, reduced postoperative complications, and shorter hospitalization in CRC surgery. Biological mechanisms were shown, (modulation of inflammatory pathways, immune activation, improved mitochondrial function, and enhanced muscle protein synthesis)	Prehabilitation should be considered a biologically active perioperative intervention. Understanding molecular mechanisms may enable development of personalized, mechanism-driven prehabilitation strategies integrated with ERAS and oncological care pathways	Available clinical evidence suggests good safety and feasibility of multimodal prehabilitation, although careful evaluation of certain immunonutrients (e.g., glutamine, arginine) is recommended due to theoretical oncologic considerations

Abbreviations: RCT—randomized controlled trial; CRC—colorectal cancer; LOS—length of hospital stay; 6 MWT—6-min walk test; VO_2_peak—peak oxygen uptake; HRQoL—health-related quality of life; HADS—Hospital Anxiety and Depression Scale; ERAS—Enhanced Recovery After Surgery; ONS—oral nutritional supplements; ASA—American Society of Anesthesiologists physical status classification; GRADE—Grading of Recommendations Assessment, Development and Evaluation; RoB—risk of bias.

## Data Availability

No new data were created or analyzed in this study. Data sharing is not applicable to this article.

## Abbreviations

The following abbreviations are used in this manuscript:

6 MWT	6-min walk test
ASA	American Society of Anesthesiologists physical status classification
CFS	Clinical Frailty Scale
CRC	colorectal cancer
ERAS	Enhanced Recovery After Surgery
GRADE	Grading of Recommendations Assessment, Development and Evaluation
HADS	Hospital Anxiety and Depression Scale
HRQoL	health-related quality of life
LOS	length of hospital stay
MA	meta-analysis
NMA	network meta-analysis
NRS-2002	Nutritional Risk Screening 2002
ONS	oral nutritional supplements
PG-SGA	Patient-Generated Subjective Global Assessment
RCT	randomized controlled trial
RoB	risk of bias
RWD	real-world data
SR	systematic review
VO_2_peak	peak oxygen uptake
